# Biomarkers and echocardiography for evaluating the improvement of the ventricular diastolic function after surgical relief of hydronephrosis

**DOI:** 10.1371/journal.pone.0188597

**Published:** 2017-11-21

**Authors:** Huei-Ming Yeh, Ting-Tse Lin, Chih-Fan Yeh, Ho-Shiang Huang, Sheng-Nan Chang, Jou-Wei Lin, Chia-Ti Tsai, Ling-Ping Lai, Yi-You Huang, Chun-Lin Chu

**Affiliations:** 1 Department of Anesthesiology, National Taiwan University College of Medicine and Hospital, Taipei, Taiwan; 2 Division of Cardiology, Department of Internal Medicine, National Taiwan University College of Medicine and Hospital Hsin-Chu Branch, Hsin-Chu, Taiwan; 3 Division of Cardiology, Department of Internal Medicine, National Taiwan University College of Medicine and Hospital, Taipei, Taiwan; 4 Department of Urology, National Chengkong University Hospital, Tainan, Taiwan; 5 Division of Cardiology, Department of Internal Medicine, National Taiwan University College of Medicine and Hospital, Yun-Lin Branch, Yun-Lin, Taiwan; 6 Insititute of Biomedical Engineering, National Taiwan University, Taipei, Taiwan; 7 Department of Anesthesiology, National Taiwan University College of Medicine and Hospital, Yun-Lin Branch, Yun-Lin, Taiwan; Scuola Superiore Sant'Anna, ITALY

## Abstract

The pathophysiology of cardio-renal syndrome (CRS) is complex. Hydronephrosis caused by urolithiasis may cause cytokine release and lead to cardiac dysfunction. The aim of this study was to evaluate cardiac function changes observed in patients who received double J placement using feasible biomarkers and echocardiography. This was a prospective, single-center study. Eighty-seven patients who presented with acute unilateral hydronephrosis and received ureteroscope stone manipulation were enrolled. Echocardiography and cytokines were measured on the day of the operation and 24 hours after the procedure. Changes before and after surgery were assessed by the paired *t*-test and Wilcoxon test. Correlation analyses between echocardiographic diastolic indices and cytokine levels were performed using Pearson’s correlation coefficients. Patients with hydronephrosis showed a higher left atrium volume index (LAVI), decreased E', and increased E/ E' ratio, which indicated diastolic dysfunction. Patients with hydronephrosis also exhibited decreased global strain rates during isovolumetric relaxation (SR_IVR_) and E/ SR_IVR_, which confirmed the diastolic dysfunction. Significant reductions in LAVI, increases in SR_IVR_ and decreases in E/ SR_IVR_ were observed after the operation. Biomarkers, such as TGF-β and serum NT-proBNP, were significantly decreased after surgery. In addition, a significant correlation was observed between the post-surgical decrease in TGF-β1 and increase in SR_IVR_. Unilateral hydronephrosis causes cardiac diastolic dysfunction, and relieving hydronephrosis could improve diastolic function. Improvements in cardiac dysfunction can be evaluated by echocardiography and measuring cytokine levels. The results of this study will inform efforts to improve the early diagnosis of CRS and prevent further deterioration of cardiac function when treating patients with hydronephrosis.

## Introduction

The human heart is vulnerable to hemodynamic changes, and both kidneys play an important role in fluid and hemodynamic regulation. As such, there are dynamic and bidirectional interactions between cardiac dysfunction and renal dysfunction. These interactions comprise the so-called cardiorenal syndrome (CRS).[1] The pathophysiology and mechanism of CRS are complex, and cytokines, including inflammatory and immunological cytokines, are thought to be major contributors to the mechanisms of disease.[2]

Urolithiasis affects approximately 5–10% of the population in industrialized countries and may cause inflammation, infection, or hydronephrosis.[3] It is the most common cause of hydronephrosis and is one of the main causes of acute or chronic kidney injury.[2] Several possible pathways have been proposed in studies of the mechanism of CRS, such as increasing inflammatory cytokines in the heart after acute kidney injury. These inflammatory cytokines may directly contribute to myocardial dysfunction.[4] However, the relationship between hydronephrosis and cardiac function has never been studied.

Acute hydronephrosis abruptly impacts kidney hemodynamics, thus affecting left ventricular (LV) function, particularly diastolic function. Furthermore, in both rats and mice, unilateral hydronephrosis has been shown to induce salt-sensitive hypertension, which may alter cardiac diastolic function.[5, 6] Therefore, we conducted this study to evaluate the changes in cardiac function that occur after relief of hydronephrosis by surgical intervention. The measurements of cardiac function were mainly based on noninvasive echocardiographic methods. By using 2-dimensional speckle tracking echocardiography, we calculated global strain and strain rates and more accurately evaluated cardiac function compared with previous measurement techniques.[7] In addition, we also measured the changes in cytokine levels to clarify the possible mechanisms associated with the hydronephrosis and CRS.

## Methods

### Ethics declaration

The protocol for this study was approved by the Institutional Ethics Review Board of National Taiwan University Hospital (Registry Number: 201205117RIC), and all subjects provided written informed consent. This study was carried out according to the International Conference on Harmonisation (ICH) / WHO Good Clinical Practice (GCP) guidelines and conformed to the principles outlined in the Declaration of Helsinki.

### Study design and subjects

This was a prospective, single-center study. Consecutive adult patients with hydronephrosis admitted to the urology ward of National Taiwan University Hospital between July 2012 and December 2012 were screened for enrollment. The exclusion criteria included a history of atrial fibrillation, valvular heart disease, chronic kidney disease, lung disease diagnosed by X-ray, and systolic dysfunction with congestive heart failure. After applying the exclusion criteria, the study population consisted of 87 patients (47 men; mean age, 56 ± 12 years). All patients presented with acute unilateral hydronephrosis caused by ureteral stones, which was diagnosed by ultrasonography or computed tomography. After enrollment, the patients underwent echocardiographic examination on the day of the operation and 24 hours after placement of the double J catheter under intravenous anesthesia with propofol infusion (120 mcg/kg/min) and bolus fentanyl (1–2 mcg/kg) adjusted according to vital sign. A 6F/7.5F semi-rigid URS was introduced into the ureter in a retrograde fashion along with a safety guide wire. Any stones were disintegrated using a Holmium:YAG laser that was operated through the URS. Ipsilateral double J stents and left indwelling Foley catheters were inserted.

Twenty control patients (12 men; mean age, 57 ± 13 years) were selected from a population of patients with benign prostate hypertrophy (BPH) who underwent elective urologic surgery. The subjects enrolled in the control group were without symptoms of heart failure and had normal ejection fractions and renal function. To comply with data privacy regulations, the personal identities of the patients were encrypted, and all data were analyzed in a de-identified manner.

### Evaluation of cardiac structure and function by echocardiography

Patients were imaged in the supine position using an ultrasound system (iE33 xMATRIX Echocardiography System, Philips Healthcare, Best, The Netherlands). Two-dimensional images were acquired with the standard parasternal and apical (apical 4-chamber, apical 2-chamber, and apical long-axis) views at a frame rate of 30 frames per second, and 3 cardiac cycles were recorded. The gains and filters were carefully adjusted to eliminate background noise and allow for a clear tissue signal. The study results were digitally stored for subsequent offline analysis. Between the 2 echocardiography evaluations, the patients’ volume statuses and hemodynamic conditions remained stable. LV end-diastolic volume (EDV), LV end-systolic volume (ESV), ejection fraction (EF), left atrium maximal volume index (LAVI), and left ventricle mass index (LVMI) were measured according to recommendations from the American Society of Echocardiography.[8] LVEDV values were calculated using the biplane method of discs (modified Simpson’s rule) in the apical 4- and 2-chamber views at end diastole, as recommended by the American Society of Echocardiography.[8] The LVEDV index (LVEDVI) was then calculated as LVEDV divided by body surface area. All Doppler values represented the average of 3 beats. Mitral flow and tissue PW-Doppler velocities were recorded in the apical 4-chamber view according to a previously described standard procedure.[9] The mitral inflow measurement included peak early (E) and peak late (A) flow velocities and the E/A ratio. During tissue Doppler imaging (TDI), a 1.5-mm sample volume was obtained at the leaflet origin of the mitral annulus.[10]

To avoid errors in tissue Doppler measurements caused by angle dependence, we maintained the angle between the PW-Doppler beam and the movement direction of the wall at < 15°. The early (E’) and late (A’) diastolic peak velocities were measured at the lateral and septal sites.

### Global strain and global strain rate

**Fig 1:**
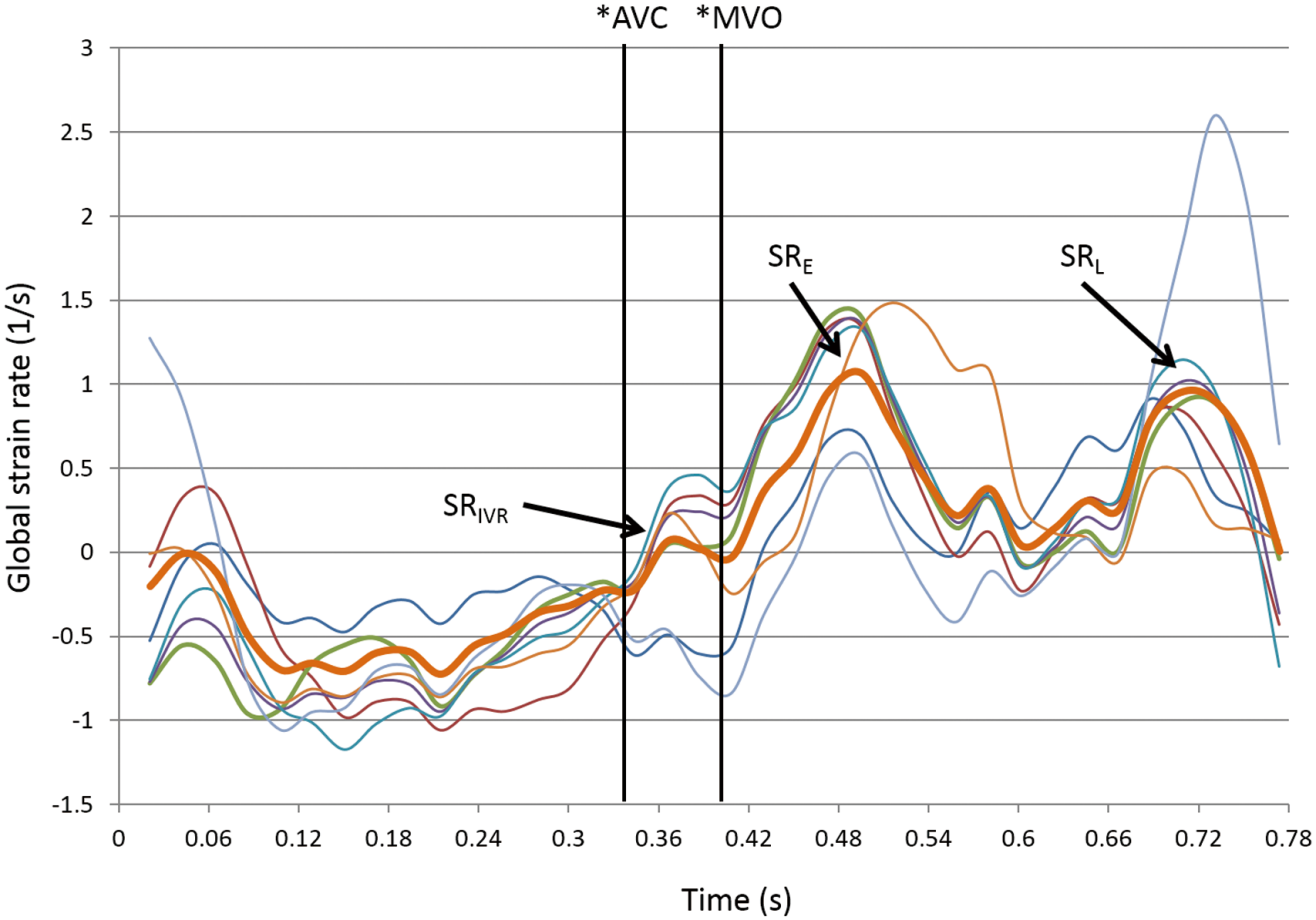
Speckle tracking, strain, and strain rate. Averaged global strain rate signal (in brown) using 2D speckle tracking. Notice the strain rate (SR) during the isovolemic relaxation time (*), early diastole phase, and late diastole phase. AVC, aortic valve closure; MVO, mitral valve opening; SR_IVR_, global SR during the isovolumetric relaxation period; SR_E_, global SR during early LV filling; SR_L_, global SR during late LV filling.

Myocardial deformation measurements were performed using tissue speckle tracking (Fig 1). Three cardiac cycles were recorded in apical 4-chamber, 2-chamber, and long-axis views at a frame rate over 80 s^-1^. In each view, a global longitudinal strain and strain rate curve were obtained and included all LV myocardial segments (6 segments per view).[11] The software package then automatically tracked the motion through the rest of the cardiac cycle (QLab 9.0, Philips). Adequate tracking was verified in real time and corrected if necessary. The LV global strain and strain rate (SR) in each view were calculated using the entire length of the LV myocardium (Fig 1). Peak global SR during the isovolumetric relaxation (IVR) period (SR_IVR_), early diastole (SR_E_), and late diastole (SR_L_) were measured. The SR values from the 3 apical views were averaged and used for final analysis.

### Reproducibility of strain rate indices

Repeat analysis of the 20 control group patients was performed by a second observer. The inter-observer correlation coefficients were 0.8 for global strain, 0.85 for SR_IVR_, 0.86 for SR_E_, and 0.67 for SR_L_. The mean differences were 1.633 ± 0.82% for global strain; 0.828 ± 0.64 s^-1^ for SR_IVR_; 0.226 ± 0.12 s^-1^ for SR_E_; and 0.154 ± 0.09 s^-1^ for SR_L_.

### Measurement of plasma and urine cytokines

Previous research has shown that serum inflammatory cytokines are involved in the mechanism of cardiac diastolic dysfunction.[12] Because of the early improvement in LV diastolic function after surgical relief of hydronephrosis, it is logical to speculate that the mechanism may be associated with the inflammatory cytokine response. Therefore, we measured serum inflammatory cytokines before and after surgical correction of hydronephrosis.

In patients with hydronephrosis, blood samples were collected from the antecubital vein with the patient in the supine position 24 hours before and after the interventions. The blood was centrifuged at 2000 *g* for 15 min, and the plasma was separated and stored at −80°C until use. Cytokines including interleukin-1β (IL-1β), tumor necrosis factor-α (TNF-α), human transforming growth factor beta (TGF-β), kidney injury molecule-1 (KIM-1), and NT-pro brain natriuretic peptide (NT-proBNP) were measured. The IL-1β (human) EIA complete kit was used for the quantitative determination of IL-1β levels in serum. The TNF-α (human) EIA kit was used for the quantitative determination of human TNF-α in serum. The TGF-β EIA enzyme immunoassay kit was used for the quantitative determination of human TGF-β in serum. The human kidney injury molecule 1 (Kim-1) ELISA Kit was used for the quantitative determination of Kim-1 concentrations in serum. The NT-proBNP (Human) ELISA Kit with standard one-step sandwich enzyme-linked immunosorbent assay technology was used to measure the concentrations of NT-proBNP in serum or urine.

Fresh urinary samples were collected and sent to the laboratory immediately after collection. After centrifugation at 2500 rpm and 4°C for 10 minutes, urinary samples were separated and stored in cryotubes at -70°C until analysis at which point they were thawed on dry ice. Before analysis, the urinary samples were centrifuged twice at 2500 rpm and 4°C for 30 minutes to avoid possible interference produced by salt precipitation in urine. The urinary biomarker NT-proBNP was assessed by ELISA kit.

### Statistical analysis

Continuous variables are presented as the mean values ± standard deviations, whereas categorical variables are presented as frequencies. Two-sample comparisons between patients with hydronephrosis and controls were performed using the *t*-test if the variables were normally distributed; the Mann-Whitney U-test was used for data that were not normally distributed. Associations between categorical variables were tested with Pearson’s chi-square test. Echocardiographic changes before and after surgery in patients with hydronephrosis were assessed by paired *t*-test. Biomarker changes were assessed by Wilcoxon signed rank test. Correlation analyses between echocardiographic diastolic indices and cytokine levels were performed using Pearson’s correlation coefficients. A p value < 0.05 was considered statistically significant. SPSS software (version 19.0, SPSS Inc., Chicago, IL, USA) was used for statistical analysis.

## Results

### Patient clinical characteristics

This study consecutively enrolled 40 women and 47 men with a median age of 56 years (range, 45–67 years) who were diagnosed with urinary stone-induced unilateral hydronephrosis and planned to undergo surgical intervention. Among the study participants, 26 patients had hypertension (29.8%), 12 had diabetes mellitus (14%), and 3 had hyperlipidemia (4.5%). There were no differences between patients with hydronephrosis and control subjects with respect to renal function, age, sex, and concomitant disease. The patients with hydronephrosis were characterized by increased body mass index (BMI), but the body surface area (BSA) was comparable between the hydronephrosis patients and controls. All of participants had normal renal function with eGFR > 90 ml/min/1.73 m^2^. The clinical characteristics of the study population and controls are summarized in Table 1.

**Table 1:** Clinical characteristics of the study population.

	Controls (n = 20)	Hydronephrosis patients (n = 87)	p-value
**Demographics**			
**Men, n (%)**	12 (50%)	47 (54)	0.628
**Age (years)**	57 (44–70)	56 (45–67)	0.836
**BMI (kg/m^2^)**	23.05 ± 4.0	25.15 ± 3.9	0.035
**BSA (m^2^)**	1.64 ± 0.1	1.62 ± 0.1	0.709
**Mean BP (mmHg)**	93 ± 11	91 ± 10	0.230
**eGFR (ml/min/1.73 m^2^)**	94.4 ± 30	91.8 ± 27	0.715
**Concomitant disease, n (%)**			
**Hypertension**	4 (20)	26 (29.8)	0.283
**Hyperlipidemia**	2 (10)	3 (4.5)	0.211
**Diabetes mellitus**	3 (15)	12 (14)	0.889
**Smoking**	7 (35)	15 (17)	0.076

BMI, body mass index; BSA, body surface area; BP, blood pressure; eGFR was estimated by MDRD formula

### Conventional and tissue Doppler echocardiography

The results of the echocardiography assessment are shown in Table 2. There were no significant differences between the patients with hydronephrosis and controls regarding various cardiac dimensions/volumes and Doppler flows, except LAVI. Patients with hydronephrosis showed significantly increased mean LAVI compared with that in controls (30.01 ± 11.1 vs. 20.8 ± 7.6 mL/m^2^, p = 0.001), which suggested the presence of dilated LA in patients with hydronephrosis probably due to cardiac diastolic dysfunction.

**Table 2:** Echocardiographic parameters of the study population.

	Control (n = 20)	Hydronephrosis patients (n = 87)		p-value	
		Before OP	After OP	Control vs. hydronephrosis	Before vs. after OP
**Heart dimensions**					
**Septum (mm)**	9.95 ± 2.0	9.53 ± 1.9	9.47 ± 1.8	0.407	0.747
**Posterior wall (mm)**	9.68 ± 1.5	9.64 ± 1.8	9.68 ± 1.5	0.933	0.873
**LVEDD (mm)**	44 ± 4.1	46 ± 5.5	46.23 ± 6.1	0.155	0.571
**LAVI (mL/m^2^)**	20.8 ± 7.6*	30.01 ± 11.1	21.88 ± 8.6^※^	0.001	< 0.001
**LVMI (g/m^2^)**	88.67 ± 19.5	93.47 ± 26.1	94.43 ± 25.8	0.442	0.728
**LVEDVI (mL/m^2^)**	42.72 ± 9.9	45.69 ± 13.19	45.40 ± 13.17	0.347	0.752
**Cardiac performance**					
**EF (%)**	71.03 ± 5.3	68.79 ± 5.8	69.14 ± 5.7	0.122	0.644
**Mitral flow**					
**E (cm/s)**	81.20 ± 19.5	76.15 ± 16.4	78.76 ± 17.7	0.09	0.646
**A (cm/s)**	75.27 ± 12.4	72.53 ± 18.0	78.16 ± 18.5^※^	0.521	0.002
**E/ A**	1.10 ± 0.3	1.05 ± 0.3	1.01 ± 0.3	0.576	0.176
**Tissue Doppler**					
**E_(medial)_ (cm/s)**	10.34 ± 2.6*	6.59 ± 2.1	6.61 ± 2.5	< 0.001	0.937
**E_(lateral)_ (cm/s)**	12.43 ± 3.1*	9.21 ± 2.8	9.36 ± 3.4	< 0.001	0.625
**E/ E_(medial)_**	8.07 ± 2.0*	11.9 ± 3.9	11.22 ± 4.4	< 0.001	0.121
**E/ E_(lateral)_**	6.78 ± 1.8*	8.68 ± 3.8	8.26 ± 3.4	0.035	0.253

*Control vs. hydronephrosis patients, p < 0.05

^※^Before vs. after the operations, p < 0.05

LVEDD, left ventricular end-diastolic diameter; LAVI, left atrium volume index; LMVI, left ventricle mass index; LVEDVI, left ventricular end-diastolic volume index; EF, ejection fraction; E/ A, the ratio of early (E) to late (A) mitral flow velocities; E_(medial)_, early diastole peak velocities of the mitral annulus at the medial site; E_(lateral)_, early diastole peak velocities of the mitral annulus at the lateral site; E/E, left ventricular filling index

We then sought to compare echocardiographic parameters related to diastolic function between those with and without hydronephrosis. Regarding the TDI indices, the mitral and lateral E’ values were significantly decreased, and the LV filling pressure index E/ E’_lateral_ was significantly increased in patients with hydronephrosis (6.78 ± 1.8 vs. 8.68 ± 3.8, p = 0.035) (Table 2). These results confirmed our conjecture that patients with hydronephrosis have impaired LV diastolic function with a resultant dilated LA.

### Global strain and strain rate

SR_IVR_, a non-invasive echocardiographic measurement, has been proposed to correlate very well with the time constant of LV relaxation (τ), which is obtained by invasive cardiac catheterization and is the gold standard measurement of cardiac diastolic function. The ratio of mitral E to SR_IVR_ (E/ SR_IVR_) also predicts LV filling pressure better than E/ E’, particularly in patients with an E/ E’ ratio of 8 to 15 and those with normal EF.[7] Herein, we also compared the LV global strain values and strain rate between patients with and without hydronephrosis. As shown in Table 3, patients with hydronephrosis had decreased strain and SR_IVR_ but increased E/ SR_IVR_, which again suggested that these patients had impaired LV relaxation and increased LV filling pressure.

**Table 3:** Strain and strain rate of the study population and changes after surgical relief of hydronephrosis.

	Control (n = 20)	Hydronephrosis (n = 87)	p-value	Before operation	After operation	p-value
**Global strain (%)**	−19.86 ± 2.5	−16.64 ± 3.2	< 0.001	−16.64 ± 3.2	−17.5 ± 3.3	0.004
**SR_IVR_ (s^-1^)**	0.402 ± 0.19	0.243 ± 0.17	< 0.001	0.243 ± 0.17	0.314 ± 0.17	< 0.001
**SR_E_ (s^-1^)**	1.319 ± 0.40	0.922 ± 0.32	< 0.001	0.922 ± 0.32	0.960 ± 0.31	0.194
**SR_L_ (s^-1^)**	1.255 ± 0.30	0.993 ± 0.24	< 0.001	0.993 ± 0.24	1.064 ± 0.26	0.018
**E/ SR_IVR_ (cm)**	393.03 ± 751	672.65 ± 148	0.030	672.65 ± 148	316.20 ± 294	0.028

SR_IVR_, strain rate during isovolumetric relaxation; SR_E_, strain rate during early diastole; SR_L_, strain rate during late diastole; E/ SR_IVR_, the ratio of mitral E to SR_IVR_

### Changes in cardiac diastolic function after surgical correction of hydronephrosis

After hydronephrosis was relieved by surgical intervention, the LAVI values significantly decreased (30.01 ± 11.1 vs. 21.88 ± 8.6 mL/m^2^, p < 0.001) and the trans-mitral A-wave values increased (72.53 ± 18.0 vs. 78.16 ± 18.5 m/s, p = 0.002) (Table 2), which indicated relief of LA stretch and improvement in LA contractile function. The change in diastolic function as evaluated by TDI was not significant (Table 2). It is possible that the diastolic function improvement was not sufficient to be detected using a conventional TDI method, and measurement of more sensitive echocardiographic parameters, such as the strain or strain rate, may be necessary.[7]

Accordingly, we found that the global strain increased (−16.64 ± 3.2 vs. −17.50 ± 3.3%, p = 0.004) (Table 3). The SR_IVR_ significantly increased (0.243 ± 0.17 vs. 0.314 ± 0.17 s^-1^, p < 0.001), and E/ SR_IVR_ significantly decreased (672.65 ± 148 vs. 316.20 ± 294 cm, p = 0.028) after surgical correction of hydronephrosis. These results indicated an improvement in diastolic function with a corresponding decrease in LV filling pressure. The strain rate during late diastole (SR_L_) also increased after surgery (0.993 ± 0.24 vs. 1.064 ± 0.26 s^-1^, p = 0.018), which was compatible with the finding of increased mitral A waves by conventional Doppler measurement.

### Changes in cytokine levels after surgical correction of hydronephrosis

**Fig 2:**
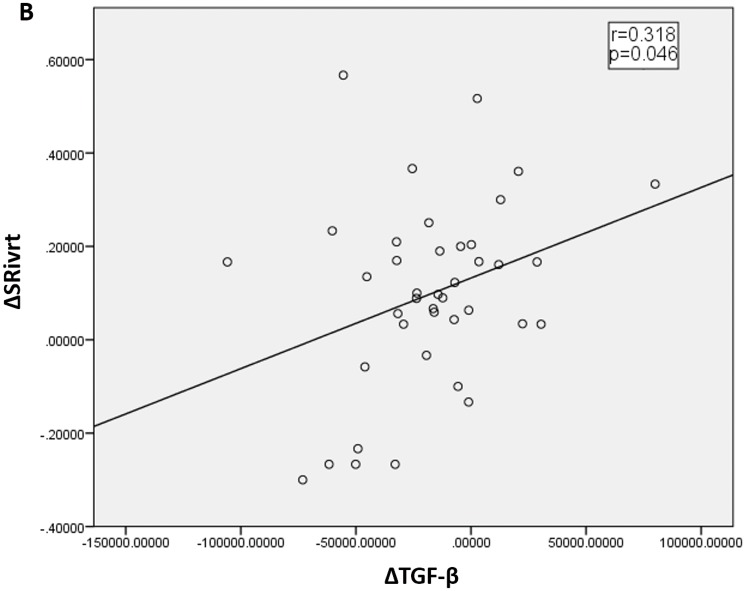
Relationship between TGF-beta levels and strain rate indices. Relationship between TGF-beta levels and strain rate indices. The post-operational changes in TGF-beta levels correlated with the post-operational changes in global strain rate during the isovolumetric relaxation period (SR_IVR_), which is a sensitive marker of cardiac diastolic function. r, correlation coefficient; P, significance level.

We found that serum TGF-β levels decreased significantly after surgical correction of hydronephrosis (Table 4). Interestingly, the change in TGF-β correlated with the change in SR_IVR_ (r = 0.319, p = 0.046; Fig 2), which is a sensitive marker of cardiac diastolic function. This result indicated that the decrease in TGF-β levels paralleled the improvement in LV diastolic dysfunction.

**Table 4:** Cytokine levels before and after surgery.

Cytokines	Before operation	After operation	p-value
**IL-1**β **(pg/mL)**	1.51 ± 2.0	1.59 ± 1.8	0.238
**TNF-**α **(pg/mL)**	6.98 ± 3.95	6.77 ± 4.89	0.257
**TGF-**β **(ng/mL)**	62.08 ± 35.89	47.47 ± 31.97	< 0.001
**KIM-1 (pg/mL)**	172.98 ± 44.825	160.94 ± 43.909	0.413
**NT-proBNP (serum, ng/ml)**	2.32 ± 0.19	1.7 ± 0.21	< 0.001
**NT-proBNP (urine, pg/mL)**	131.01 ± 2.36	134.28 ± 2.54	< 0.001

IL-1β, interleukin-1 beta; TNF-α, tumor necrosis factor alpha; TGF-β, human transforming growth factor beta; KIM-1, kidney injury molecule-1; NT-proBNP, NT-pro brain natriuretic peptide

### Changes in serum and urine NT-proBNP levels after surgical correction of hydronephrosis

In addition to the positive findings mentioned above, we further measured both serum and urine NT-proBNP levels in patients with hydronephrosis before and after operation. Interestingly, the NT-proBNP levels were significantly lower after surgery (2.32 ± 0.19 vs. 1.7 ± 0.21, p < 0.001) (Table 4). In addition, the urine level of NT-proBNP was significantly increased after surgical correction (131.01 ± 2.36 vs. 134.28 ± 2.54, p < 0.001) (Table 4).

## Discussion

The main findings of the present study are as follows: 1) Compared with controls, patients with hydronephrosis had decreased E’ and SR_IVR_, increased LV filling indices (E/ E’ and E/ SR_IVR_) and higher LAVI, which suggested diastolic dysfunction with a dilated left atrium; 2) Surgical relief of hydronephrosis resulted in improved LV relaxation, which was reflected by the changes in SR_IVR_ and E/ SR_IVR_; 3) Patients with hydronephrosis exhibited reduced serum TNF-α, TGF-β, and KIM-1 levels after surgical relief of hydronephrosis. In addition, the changes in TGF-β levels were correlated with the changes in SR_IVR_, which is a sensitive marker of ventricular diastolic function (a surrogate of ventricular time constant [τ]).[7] 4) After double J catheter placement, patients with hydronephrosis had decreased NT-proBNP levels in the blood and increased NT-proBNP levels in the urine.

Cardiac dysfunction can cause renal dysfunction and vice versa through multiple mechanisms. In type 3 and type 4 CRS, which are characterized by renal dysfunction leading to cardiac dysfunction, many possible pathophysiological mechanisms have been proposed. These mechanisms include volume expansion, drop in glomerular filtration rate, sympathetic activation, renin-angiotensin-aldosterone system activation, vasoconstriction, electrolyte imbalance, and inflammatory and humoral factors.[13] Thus, the relationship between hydronephrosis and cardiac function in our study may be a “subclinical” form of type 3 or type 4 CRS because none of the patients had clinical symptoms of heart failure and all had normal renal function. Therefore, our study established for the first time the possibility that unilateral hydronephrosis can cause cardiac diastolic dysfunction, which represents a subclinical form of CRS or “cardioureteral syndrome”. We have also first established the possibility of diastolic dysfunction during the very early stages of renal disorder.

Impaired LV relaxation and increased LV diastolic stiffness are the major causes of diastolic dysfunction that lead to elevated LV filling pressure and LA stretch. Diagnostic evidence of diastolic dysfunction can be obtained noninvasively by tissue Doppler (E/ E’ > 15), trans-mitral inflow velocities (mitral E/ A ratio), and plasma levels of natriuretic peptides (BNP).[14] However, there are several drawbacks to these methods. For example, the E/A ratio is pre-load dependent, and it has been reported that E/ E' does not provide accurate noninvasive measurements of LV filling pressure.[15] Therefore, in this study, we did not observe a significant change in E/ E’ values or mitral E/ A ratios after surgical relief of hydronephrosis. However, we did find a significant decrease in LAVI, which suggested relief of LA stretch and improvement in LV diastolic dysfunction.

It was recently demonstrated that E/SR_IVR_ could predict LV filling pressure more precisely than E/ E’, and SR_IVR_ has a better correlation with the time constant of LV relaxation (τ) when measured invasively.[7] In agreement with this study, in addition to decreased LA volume, we also observed significantly decreased E/ SR_IVR_ and increased SR_IVR_ after surgical correction of hydronephrosis, which reflected the decreased LV filling pressure and improved LV diastolic function.

In contrast to SR_E_, SR_L_ was not related to LV relaxation and compliance.[16] During atrial systole, the ventricle is passive, and SR_L_ may reflect the combined result of LV passive recoil process and left atrial “kick”.[17] In the current study, the increase in both late mitral inflow velocities (A wave) and SR_L_ implied increased atrial function after surgery, probably resulting from relief of LA stretch (caused by diastolic dysfunction), as reflected by decreased LAVI.

Because of the early improvement in LV diastolic function and performance after surgical relief of hydronephrosis, it is logical to speculate that the mechanism may occur through hormonal or inflammatory responses. Accordingly, we found a corresponding decrease in serum TGF-β levels after surgical correction of hydronephrosis by double J insertion.

TGF-β is a multifunctional peptide that plays a central role in regulating extracellular matrix formation and cell matrix adhesion processes. It has been shown that TGF-β plasma levels increase in rats with unilateral ureteral obstruction and in children with congenital obstructive uropathy.[18] TGF-β also modulates the expression of the calcium-handling protein, sarcoendoplasmic reticulum calcium transport ATPase 2a (SERCA2a). SERCA2a is responsible for refilling calcium into the sarcoplasmic reticulum during the diastolic phase of a heart cycle, and decreased SERCA2a expression results in diastolic dysfunction.[19] In addition, in the animal model, increased TGF-β levels activate the phosphorylation of Smad2, which upregulates Nox4-based NADPH oxidase and causes SERCA oxidation and dysfunction.[20] These results suggest that elevated TGF-β causes diastolic dysfunction in the scenarios of ureteral obstruction and hydronephrosis.

TGF-β also plays a critical role in myocardial fibrosis, which also causes diastolic dysfunction. Fernando et al. showed that unilateral nephrectomy causes early cardiac apoptosis, fibrosis, and diastolic dysfunction in rats.[21] Therefore, chronic hydronephrosis may result in cardiac dysfunction by fibrosis.

Our patients were in the early stages of unilateral hydronephrosis without kidney injury. However, at this early stage, cardiac diastolic dysfunction could be been detected. In addition, we found that TGF-β levels paralleled the changes in E/ SR_IVR_ after surgery and TGF-β may be the underlying cytokine involved in hydronephrosis-related subclinical CRS.

B-type natriuretic peptide (BNP) and its cleavage fragment, NT-proBNP, are known to be influenced by preload and ventricular abnormalities and are good markers of myocardial damage and stress in patients with renal failure.[22] Compared with BNP whose clearance occurs in the kidneys and other organs, NT-proBNP is only cleared by the renal system.[23] In addition, in patients with heart failure, renal disorders are confirmed by depletion of plasma NT-proBNP. [24] Therefore, NT-proBNP could be more suitable than BNP when evaluating the correlation between cardiac function and renal function in patients with CRS.

From this study, we found satisfactory agreement that patients with hydronephrosis had decreased plasma NT-proBNP levels and increased urine NT-proBNP levels after effective relief of hydronephrosis. This change is rapid and could be detected soon after surgery either by serum or urine analysis of NT-proBNP. Based on these findings, we concluded that NT-proBNP is a helpful, inexpensive, simple, and reliable biomarker for reflecting the clinical condition of patients with subclinical CRS.

In summary, we proved that surgical correction of hydronephrosis can improve diastolic function. Patients with unilateral hydronephrosis had reduced levels of serum TGF-β, reduced levels of blood NT-proBNP and increased NT-proBNP clearance in the urine. Relieving hydronephrosis resulted in improved LV relaxation, decreased LV filling pressure, decreased LA volume, and enhanced LA function with a corresponding reduction in serum TGF-β levels. Our study implied a strong association between biochemical markers and echocardiographic abnormalities. According to these results, we can improve efforts for the early diagnosis of CRS and prevent further deterioration of cardiac or renal function when treating patients with hydronephrosis.

## Supporting information

S1 Table Echocardiographic parameters before and after operation. (DOCX)

S2 Table Strain and strain rate before and after operation. (DOCX)

S3 Table Cytokines before and after operation. (DOCX)

S4 Table STROBE statement checklist. (DOCX)
