# Supplementary material for: Biomarkers and echocardiography for evaluating the improvement of the ventricular diastolic function after surgical relief of hydronephrosis
Source: PLoS One. 2017 Nov 21;12(11):e0188597. doi: 10.1371/journal.pone.0188597 (PMC5697892; doi:10.1371/journal.pone.0188597)
Supplement: S1 Table — (DOCX) [file pone.0188597.s001.docx]

**S1 Table Echocardiographic parameters before and after operation**

| Hydronephrosis (n=87) | Before URS-SM | After URS-SM |
| --- | --- | --- |
| **Heart dimensions** |  |  |
| **Septum (mm)** | 9.53 ± 1.9 | 9.47 ± 1.8 |
| **Posterior wall (mm)** | 9.64 ± 1.8 | 9.68 ± 1.5 |
| **LVEDD (mm)** | 46 ± 5.5 | 46.23 ± 6.1 |
| **LAVI (mL/m^2^)** | 30.01 ± 11.1 | 21.88 ± 8.6 |
| **LVMI (g/m^2^)** | 93.47 ± 26.1 | 94.43 ± 25.8 |
| **LVEDVI (mL/m^2^)** | 45.69 ± 13.19 | 45.40 ± 13.17 |
| **Cardiac performance** |  |  |
| **EF (%)** | 68.79 ± 5.8 | 69.14 ± 5.7 |
| **Mitral flow** |  |  |
| **E (cm/s)** | 76.15 ± 16.4 | 78.76 ± 17.7 |
| **A (cm/s)** | 72.53 ± 18.0 | 78.16 ± 18.5 |
| **E/ A** | 1.05 ± 0.3 | 1.01 ± 0.3 |
| **Tissue Doppler** |  |  |
| **E_(medial)_ (cm/s)** | 6.59 ± 2.1 | 6.61 ± 2.5 |
| **E_(lateral)_ (cm/s)** | 9.21 ± 2.8 | 9.36 ± 3.4 |
| **E/ E_(medial)_** | 11.9 ± 3.9 | 11.22 ± 4.4 |
| **E/ E_(lateral)_** | 8.68 ± 3.8 | 8.26 ± 3.4 |
