# Supplementary material for: Biomarkers and echocardiography for evaluating the improvement of the ventricular diastolic function after surgical relief of hydronephrosis
Source: PLoS One. 2017 Nov 21;12(11):e0188597. doi: 10.1371/journal.pone.0188597 (PMC5697892; doi:10.1371/journal.pone.0188597)
Supplement: S2 Table — (DOCX) [file pone.0188597.s002.docx]

**S2 Table Strain and strain rate before and after operation**

| Hydronephrosis (n = 87) | Before URS-SM | After URS-SM |
| --- | --- | --- |
| **Global strain (%)** | −16.64 ± 3.2 | −17.5 ± 3.3 |
| **SR_IVR_ (s^-1^)** | 0.243 ± 0.17 | 0.314 ± 0.17 |
| **SR_E_ (s^-1^)** | 0.922 ± 0.32 | 0.960 ± 0.31 |
| **SR_L_ (s^-1^)** | 0.993 ± 0.24 | 1.064 ± 0.26 |
| **E/ SR_IVR_ (cm)** | 672.65 ± 148 | 316.20 ± 294 |
