# Supplementary material for: Biomarkers and echocardiography for evaluating the improvement of the ventricular diastolic function after surgical relief of hydronephrosis
Source: PLoS One. 2017 Nov 21;12(11):e0188597. doi: 10.1371/journal.pone.0188597 (PMC5697892; doi:10.1371/journal.pone.0188597)
Supplement: S3 Table — (DOCX) [file pone.0188597.s003.docx]

**S3 Table Cytokines before and after operation**

| Hydronephrosis (n= 87) | Before URS-SM | After URS-SM |
| --- | --- | --- |
| **IL-1**β **(pg/mL)** | 1.51 ± 2.0 | 1.59 ± 1.8 |
| **TNF-**α **(pg/mL)** | 6.98 ± 3.95 | 6.77 ± 4.89 |
| **TGF-**β **(ng/mL)** | 62.08 ± 35.89 | 47.47 ± 31.97 |
| **KIM-1 (pg/mL)** | 172.98 ± 44.825 | 160.94 ± 43.909 |
| **NT-proBNP (serum, ng/ml）** | 2.32 ± 0.19 | 1.7 ± 0.21 |
| **NT-proBNP (urine, pg/mL）** | 131.01 ± 2.36 | 134.28 ± 2.54 |
